# 

**Published:** 1897-01

**Authors:** 


Vol. XVIII. JANUARY, 1897. No. 1.
PUBLISHED MONTHLY AT $3 A YEAR. SINGLE COPIES, 30 CENTS.

### THE JOURNAL OF COMPARATIVE MEDICINE AND VETERINARY ARCHIVES

EDITED AND PUBLISHED BY
RUSH SHIPPEN HUIDEKOPER, Veterinarian (Alfort),
W. HORACE HOSKINS, D.V.S.,
H. D. GILL, V.S.

### UNITED STATES VETERINARY MEDICAL ASSOCIATION.

**Proceedings of the Thirty-third Annual Meeting held at Buffalo, N. Y., September, 1896. (Continued.)**

The Relations of Horseshoers to Veterinarians and Veterinary Colleges. (*Continued.*)
By H. D. GILL, V.S.. . . . . . . . . . . . . . . . . 97
Discussion on Tuberculosis . . . . . . . . . . . . . . 106

ALL COMMUNICATIONS AND PAYMENTS SHOULD BE ADDRESSED TO
**Dr. W. Horace Hoskins, Managing Editor, 3452 Ludlow St., Philadelphia, Pa.**
Copies may be had on order of all news-dealers. In London of Baillière, Tindall & Cox.

